# Patterns and Complications of Congenital Heart Disease in Adolescents and Adults in Ethiopia

**DOI:** 10.3390/jcdd11080253

**Published:** 2024-08-19

**Authors:** Misikr Alemu Eshetu, Dejuma Yadeta Goshu, Molla Asnake Kebede, Hashim Meketa Negate, Abiel Berhe Habtezghi, Paula Marsh Gregory, Amenu Tolera Wirtu, Jickssa Mulissa Gemechu

**Affiliations:** 1 Department of Internal Medicine, School of Medicine, College of Medicine and Health Science, Mizan-Tepi University, Mizan-Aman P.O. Box 260, Ethiopia; misikralemu@gmail.com (M.A.E.); mollaasnake75@gmail.com (M.A.K.); 2 Department of Cardiology, College of Health Sciences, Addis Ababa University, Addis Ababa P.O. Box 9086, Ethiopia; dejuya@yahoo.com; 3 Department of Radiology, School of Medicine, College of Medicine and Health Science, Mizan-Tepi University, Mizan-Aman P.O. Box 260, Ethiopia; hashimemeketa@gmail.com; 4 Mercy Catholic Medical Center, Darby, PA 19023, USA; abielberhe7@gmail.com; 5 Meritus Medical Center, Meritus School of Osteopathic Medicine, Hagerstown, MD 21742, USA; paula.gregory@meritushealth.com (P.M.G.); amannut2002@gmail.com (A.T.W.); 6 Department of Foundational Medical Studies, Oakland University William Beaumont School of Medicine, Rochester, MI 48309, USA

**Keywords:** atrial septal defect, congenital heart disease, ventricular septal defect, Ethiopia cardiac clinic

## Abstract

Background: Congenital heart disease (CHD) encompasses morphofunctional anomalies in the heart and circulatory system present at birth, which may not become apparent until later in life. In Ethiopia, there needs to be more understanding of the prevalence, patterns, and associated complications of CHD malformations. This study aimed to investigate the patterns and complications of CHDs among patients receiving follow-up care at a specialized university referral hospital in Ethiopia. Methods: A hospital-based cross-sectional study was conducted on 199 patients with CHDs to assess the patterns and complications of defects. Retrospective data were collected from 16,972 patients who had follow-ups at a cardiac clinic in 2021 using medical records, and a statistical analysis was performed with SPSS version 24. Results: The most prevalent types of CHDs in our study population were atrial septal defects (ASDs) at 41.2% (82 cases), ventricular septal defects (VSDs) at 26.6% (53 cases), and patent ductus arteriosus (PDAs) at 9.5% (19 cases). Complications related to CHDs were observed in 69.3% (138) of patients, with 30.7% (61) experiencing a single complication and 39.2% (87) experiencing multiple complications. Conclusion: This study found a higher prevalence of CHDs in females (77.8%) compared to males, a trend consistent across various atrial and ventricular defect types. Individuals aged 15 to 25 years exhibited the highest incidence of ASD and VSD. Moreover, CHD-related anomalies were present in 69.3% of the patients studied.

## 1. Introduction

Congenital heart disease (CHD) manifests as anomalies in the cardio-circulatory structure or function present at birth, though they may remain undetected for years. Typically arising from aberrant fetal development, CHDs lead to anatomical irregularities and subsequent abnormal blood flow patterns [1]. CHDs are generally categorized into simple, intermediate, and complex malformations [2,3]. A commonly used secondary classification scheme subdivides CHDs into groups such as cyanotic heart disease, malformations with short circuits, and valvular lesions [4].

The most prevalent CHDs are atrial septal defects (ASDs), ventricular septal defects (VSDs), patent ductus arteriosus (PDAs), coarctations of the aorta, Tetralogy of Fallot (TOF), transpositions of the great artery, pulmonary stenosis (PSs), and aortic stenosis—altogether, these conditions account for 90% of CHD cases [3,5,6]. Various factors, including genetic and environmental influences, contribute to CHDs during development. Single heterogeneous gene (NKX2.5) mutations and chromosomal aberrations (where a piece of the chromosome is missing or present in duplicate) account for 10% of all cardiac abnormalities [2]. Maternal diabetes, rubella infection during pregnancy, exposure to thalidomide and isotretinoin in early pregnancy, and maternal alcohol consumption exceeding one drink per day are the primary environmental risk factors [2,7].

Complications of CHDs include heart failure, cyanosis, pulmonary hypertension, Eisenmenger’s syndrome, and cardiac arrhythmias. However, these consequences are rare in Western countries due to advancements in corrective procedures, though they remain prevalent in developing countries [1].

CHDs, affecting 0.8% to 2% of global live births, represent the most common congenital birth defects [8,9,10,11]. In 2013, Africa accounted for 23% of global CHD prevalence by continent. Seven nations—Nigeria, Ethiopia, the Democratic Republic of Congo, Egypt, Tanzania, Uganda, and Kenya—accounted for over half (51.4%) of the prevalence. Ethiopia, second only to Nigeria, reported 23,866 CHD cases annually [12,13,14].

Understanding the prevalence and types of CHDs in the Ethiopian population is essential for assessing the disease’s burden. This knowledge is crucial for public health planning and resource allocation. Identifying the most common CHDs in Ethiopia makes it possible to develop screening programs for early diagnosis. Early detection facilitates timely interventions, improving affected children’s outcomes and quality of life. Studying CHDs in Ethiopia can also provide insights into the genetic and environmental factors contributing to the condition, leading to a better understanding of its etiology and the development of targeted prevention strategies. Additionally, investigating CHDs in Ethiopia can help researchers evaluate its impact on infant and child mortality and morbidity. This data is vital for setting maternal and child cardiac health program priorities.

Studying CHDs in Ethiopia can facilitate international collaborations and the sharing of best practices. This can lead to improved health outcomes for children with CHDs and contribute to the overall strengthening of the country’s cardiac healthcare system.

Therefore, this study seeks to explore the patterns and complications of CHDs in Ethiopia by examining them through clinical and epidemiological perspectives on diseases.

## 2. Materials and Methods

This research was carried out at the cardiac referral clinic of Tikur Anbessa Specialized Hospital (TASH). This hospital-based cross-sectional study was conducted on 199 patients with valid cases of adult CHDs. This study focused on age at the time of diagnosis of patients with CHDs who attended the cardiac referral clinic from 1 January to 30 December 2021, at TASH.

Total population sampling method was utilized, including all cases of CHDs. Retrospective data were collected from 16,972 patients who had follow-ups at a cardiac referral clinic. Only patients over the age of 15 with complete medical records pertinent to this study, and who were diagnosed with CHDs through echocardiography (ECG) by a cardiologist were included. 

The data collection checklist was adapted from various comparable studies and tailored to align with the current CHD management and diagnostic protocols. Nurses and residents at the cardiac referral clinic conducted the data collection. The checklist includes socio-demographic information, symptoms and signs, significant risk factors, ECG and chest X-ray (CXR) results, and relevant laboratory findings extracted from patient charts.

Data collectors received training focused on this study’s objectives and data collection procedures. The principal investigator closely monitored and evaluated the data collection process to ensure the consistency and completeness of the data.

The data were initially entered into Microsoft Excel 2013 (Microsoft Corporation, Redmond, WA, USA) and subsequently transferred to SPSS version 24 (IBM Corp., Armonk, NY, USA) for analysis. Descriptive statistics were used to examine socio-demographic traits and CHD patterns. The correlation between each CHD type and associated malformations was assessed through cross-tabulation. Statistical significance was determined using a *p*-Value threshold of <0.05.

## 3. Results

**Figure 1:**
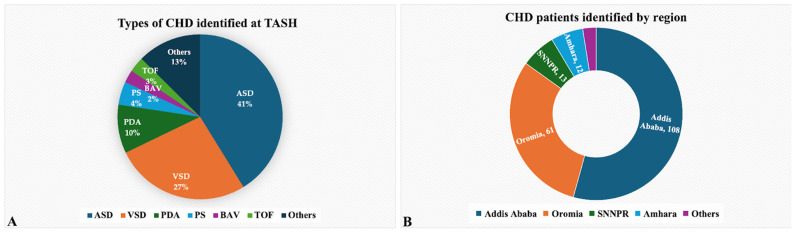
Distribution of Congenital Heart Diseases (CHD) at Tikur Anbessa Specialized Hospital (TASH). (**A**) shows types of CHDs identified at TASH. (**B**) shows CHD patients identified by region.

A total of 199 patient records were assessed at the TASH cardiac referral clinic, identifying valid cases of CHDs. The patients’ ages ranged from 15 to 74 years, with the majority falling between 15 and 25 years old (97 individuals, representing 48.7% of the sample) [Table 1]. Most of the patients were female, totaling 155 individuals (77.8%) [Figure 1A]. Patient distribution by region was identified: one hundred and eight patients (54.3%) were from Addis Ababa, sixty-one (30.7%) were from Oromia, thirteen were from Southern Nation and Nationalities People’s Region (SNNPR), twelve were from Amhara, and the remaining five were from other regions of Ethiopia [Figure 1B].

### 3.1. Family History and Presence of Associated Congenital Anomalies

No identifiable risk factors were present in any of the patients, and none had a family history of cardiac issues. Congenital syndrome was detected in seven patients (3.5%), with Down Syndrome being the most identified, occurring in six of these cases (3.0%).

### 3.2. Prevalence of Congenital Heart Diseases

In this study, the most prevalent subtype of adult CHD identified was ASDs comprising approximately 41.2% (82) of cases. Following ASDs, VSDs and PDAs were notable, contributing to 26.6% (53) and 9.5% (19) of cases, respectively. PSs accounted for 4% (10) of cases, while Bicuspid Aortic Valves (BAVs) was observed in 2.5% (5) of patients. TOF was diagnosed in six patients (3.01%). The remaining 12.6% cases (25) were classified as other complications, representing a variety of different adult CHD types [Figure 1]. Notably, among patients in the other complication group, 52% (13) had combined congenital heart disease, indicating multiple types of CHDs [Table 1].

### 3.3. Patterns of Congenital Heart Diseases by Age and Their Association

This study on the age distribution of CHDs unveiled notable findings. ASDs were most prevalent in the 15–25 age group, constituting 45% of cases, followed closely by the 26–40 age group with 41.46% of cases. In contrast, the occurrence of ASDs in older age patients was notably lower, with 12% in the 41–60 group and merely 1.2% in individuals aged 60 and above. VSDs primarily affected individuals in the 15–25 and 26–40 age groups, comprising 50.9% and 41.5% of cases, respectively. The age distribution for PDAs and BAVs was also concentrated in the 15–25 age range, each representing half of the cases. Moreover, the analysis found no significant correlation between age at initial diagnosis of CHD and its pattern, with a *p*-Value of 0.64 indicating no association [Table 1].

### 3.4. Patterns of Congenital Heart Diseases by Sex and Their Association

Among patients referred to cardiac clinics, females constituted 77.8% (155) of cases of CHDs. Females exhibited a higher prevalence across various types of CHDs: 77.35% (41) of VSDs, 84.2% (16) of PDAs, 82.9% (68) of ASDs, and 77.78% (7) of PSs. Conversely, VSDs accounted for 31.8% (14) of cases among male patients. The association between gender and CHD pattern showed no significant correlation, with a *p*-Value of 0.242 [Table 2].

### 3.5. Identified Congenital Heart Disease-Related Malformations and Their Association

Complications were identified in 69.3% (138) of the patients. Within this group, 49.3% (68) experienced a single complication, while 50.7% (70) encountered multiple complications. Among those with a complication, the predominant type was heart failure, observed in 27.5% (38) cases, followed by polycythemia in 9.4% (13) cases, and pulmonary hypertension in 8.7% (12) cases [Table 3]. Among patients with multiple complications, the most prevalent combination was heart failure and pulmonary hypertension, affecting 26.8% (37) cases, followed by heart failure combined with pulmonary hypertension and cardiac arrhythmias, noted in 6.5% (9) cases, and pulmonary hypertension along with arrhythmias in 5.7% (7) cases [Table 3]. Upon analyzing the correlation between complications and the type of congenital disease, the *p*-Value obtained was 0.28, indicating no significant association [Table 3].

### 3.6. Surgical Intervention

Out of the entire patient population, 14.57% (29) of cases underwent surgery. Among them, 96.55% patients (28) had one surgical procedure, while 3.45% (1) patient underwent two surgeries. ASD closure emerged as the most frequent procedure, constituting 55.17% (16) cases, followed by PDA ligation in 13.79% (4) cases. Additionally, among the patients who did not undergo surgical intervention initially, 64 cases are scheduled for future surgical management.

## 4. Discussion

CHDs in Ethiopia present unique challenges and considerations compared to other countries due to various factors such as the healthcare infrastructure, socio-economic conditions, and public health awareness. The prevalence of CHDs in Ethiopia is not well documented due to limited developmental, clinical, and epidemiological studies. However, hospital-based studies suggest that CHDs are a significant cause of pediatric morbidity and mortality. The most common types of CHDs observed in Ethiopia include VSDs, ASDs, and TOF. Rheumatic heart disease, though not congenital, also remains a significant concern due to its prevalence and impact on pediatric heart health.

The predominant CHDs identified in this study were ASDs, VSDs, and PDAs, which collectively accounted for the majority of cases. This distribution aligns with findings from studies conducted in Canada and Taiwan, indicating consistent prevalence patterns across different populations [15,16]. Notably, females constituted a significant proportion of CHD cases in this study, consistent with observations from Canada and Taiwan [16,17,18]. The high prevalence of ASDs and VSDs observed in this study mirrors findings from a meta-analysis conducted in East African countries and a study in Ethiopia, underscoring the regional consistency in CHD profiles [17,19,20].

The occurrence of multiple CHDs in a single patient was a notable finding in this study, with a higher prevalence compared to studies in Mexico and China [14,16]. This finding suggests a potentially unique aspect of CHD manifestation in this population. Regarding age distribution, a peak in CHD cases was observed in the 15–25 age group, which is consistent with findings from Mexico and Taiwan. Female predominance across various types of CHDs echoes findings from Taiwan, suggesting potential age and gender-related differences in CHD prevalence [4,15,17]. Many regions in Ethiopia lack access to advanced diagnostic tools like ECG, which are crucial for the early detection and accurate diagnosis of CHDs. Due to limited cardiac healthcare infrastructure, many cases of CHDs are diagnosed late at referral hospital or remain undiagnosed until complications have already developed, reducing the chances of effective intervention.

Multiple complications were prevalent among CHD patients in this study, with a significant proportion experiencing heart failure, pulmonary hypertension, cardiac arrhythmias, and polycythemia. The high complication rate observed here exceeds rates reported in other studies in Ghana and Tanzania [8,9,14]. This discrepancy may be attributed to the referral nature of the study center, wherein complicated cases are likely to be concentrated, leading to a higher observed complication rate. Access to cardiac surgery is limited in Ethiopia, where only a few specialized centers in urban areas like Addis Ababa can perform complex cardiac surgeries. Many patients require referrals abroad for advanced surgical care. The medical management of CHDs with therapeutics is more common but often inadequate due to the lack of specialized pediatric cardiologists and consistent access to necessary cardiovascular drugs.

This study provides valuable insights into the patterns and complications of CHDs in the study population, reaffirming patterns observed in other countries while highlighting potential unique aspects of CHD manifestation in the Ethiopian context. Further research is warranted to explore the underlying factors contributing to the observed variations in CHD prevalence and complications across different populations by age and gender. 

The cost of diagnosing and treating CHDs is prohibitive for many Ethiopian families, particularly those from rural areas. This economic barrier often leads to delays in seeking cardiac care and higher mortality rates. Awareness about CHDs among the general population is also low. This lack of awareness can result in delayed cardiac healthcare-seeking behavior and missed opportunities for early intervention. Various non-governmental organizations and international health bodies are involved in efforts to improve CHD care in Ethiopia, including providing training, resources, and funding for treatment programs. Indeed, the consequences of congenital heart conditions should not be underestimated, as they have a substantial impact on patient-centered hospital care and global health outcomes.

## 5. Conclusions

Tackling CHDs in Ethiopia necessitates a comprehensive strategy that involves enhancing healthcare infrastructure, raising public awareness, and training more health professionals. Our study found that females are more affected by CHDs than males, with most types of CHDs being more prevalent in females. VSDs, PDAs, and ASDs are the most common types of CHDs, with ASDs and VSDs being especially prevalent in the 15–25 age group, along with half of all PDAs and BAVs. Complications were identified in nearly 70% of patients, with over half experiencing multiple complications. Our study suggests conducting multi-center or community-based prevalence studies to understand Ethiopia’s national landscape of CHDs better. Additionally, this study recommends improving prenatal and childhood screening for congenital anomalies and enhancing surgical intervention capabilities to ensure children with CHDs receive necessary treatment and follow-up. This entails establishing more well-equipped healthcare facilities like cardiac rehabilitation centers. In summary, our study of CHDs at TASH is crucial for improving patient care, advancing cardiac knowledge, and enhancing cardiac health strategies in Ethiopia.

This study is limited by its small sample size, which reflects the trends of CHDs in our setting. Additionally, it was a single-center investigation that included all patients visiting TASH from various regions across Ethiopia.

## Figures and Tables

**Table 1:** Distribution of congenital heart disease (CHD) types by age identified at Tikur Anbessa Specialized Hospital (TASH). No significant correlation was found between age at initial diagnosis of CHDs and its pattern, with a *p*-Value of 0.64.

Patient Age	Types of Congenital Heart Disease								*p*-Value
	%	ASD	VSD	PDA	PS	Others One	Others Two or More	Total	
15–25	48.70	37	27	10	4	11	8	97	0.64
26–40	38.20	34	22	6	4	5	5	76	
41–60	12.06	10	3	3	2	6	0	24	
>60	1.04	1	1	0	0	0	0	2	
Total	100	82	53	19	10	22	13	199	

**Table 2:** Distribution by gender of congenital heart disease (CHD) and their association at Tikur Anbessa Specialized Hospital (TASH).

		Types of Congenital Heart Disease							Total	*p*-Value
		ASD	VSD	PDA	PS	BAV	TOF	Others		
Patient gender	Female	68	41	16	7	3	5	15	155	0.242
	Male	14	12	3	2	2	1	10	44	
Total		82	53	19	9	5	6	25	199	

**Table 3:** Types of complications associated with congenital heart diseases (CHDs) identified at Tikur Anbessa Specialized Hospital (TASH).

Complications	Frequency	%	Association with the Type of CHD *p*-Value
Heart failure	38	27.5	0.28
PH	12	8.7	
Polycythemias	13	9.4	
Cardiac Arrhythmias	3	2.2	
Venous thrombosis	2	1.4	
Heart failure with Eisenmenger’s	1	0.7	
Heart failure with PH	37	26.8	
Heart failure with arrhythmias	5	3.6	
Heart failure, PH, and arrhythmias	9	6.5	
PH and Arrhythmias	7	5.1	
Heart failure and polycythemia	6	4.3	
Others	5	3.6	
Total	138	100.0	

## Data Availability

The data that support the findings of this study were obtained from patient records and contain confidential personal information. Due to privacy and ethical considerations, these data are not publicly available. However, access to the data may be permitted under certain conditions, in compliance with ethical guidelines and regulations. Interested researchers can reach out to the cardiac referral clinic at Tikur Anbessa Specialized Hospital (TASH), Addis Ababa University for more information.
